# Guillain-Barré-Like Syndrome From Esophageal Squamous Cell Carcinoma

**DOI:** 10.7759/cureus.26158

**Published:** 2022-06-21

**Authors:** Aryanna Jordan, Alena Makarova, Jacob J Adashek

**Affiliations:** 1 Internal Medicine, University of South Florida, H. Lee Moffitt Cancer Center and Research Institute, Tampa, USA; 2 Neurology, University of South Florida, Tampa, USA

**Keywords:** guillain-barré-like syndrome, intravenous immunoglobulin, platinum chemotherapy, squamous cell cancer, esophageal cancer

## Abstract

Guillain-Barré-like syndrome (GBS) has been noted in solid tumors such as hepatocellular carcinoma, gastric, breast, and colon cancer and is characterized by an ascending weakness generally with mild paresthesias; however, it has not been noted in esophageal squamous cell carcinoma. A 64-year-old woman with moderately differentiated squamous cell carcinoma of the upper esophagus receiving 5-fluorouracil and cisplatin chemotherapy concurrently with radiation therapy presented for two weeks of progressive lower extremity weakness and numbness. She experienced acute worsening of symptoms after her cisplatin infusion. She underwent a lumbar puncture that revealed findings consistent with GBS and she began treatment with intravenous immunoglobin. She had gradual improvement in her weakness and paresthesias and was discharged to an inpatient rehabilitation facility. This case documents the first noted association between GBS and esophageal squamous cell carcinoma.

## Introduction

In 2020, there were 604,000 new cases of esophageal cancer in the United States [1]. This type of cancer is sixth in overall mortality and is more common in men [1]. The two most common subtypes are squamous cell carcinoma (SCC) and adenocarcinoma. Esophageal squamous cell carcinoma (ESCC) accounts for 90% of all esophageal cancer cases [1]. Eastern Asia exhibits the highest incidence of esophageal cancer followed by Africa, Northern Europe, and South-Central Asia [1]. The etiology of ESCC in high-risk areas is in decline which is thought to be secondary to dietary improvements [1]. The most common risk factors in other parts of the world are alcohol and tobacco use [1]. Although the five-year survival rate of ESCC is improving, it is largely based on increased diagnosis at early stage of disease. Patients with localized and regional stages had overall increase in five-year survival rate of 21.1%; however, the five-year survival trends for patients with distant stage cancer were unable to be calculated based on low sample size [2]. Previous studies have indicated that the median overall survival in patients with metastatic ESCC ranges between four and six months [3]. 

Guillain-Barré-like syndrome (GBS) is the most common cause of paralysis worldwide [4]. It presents as an ascending paralysis that can spread proximally in days. It can be characterized by peripheral neuropathy and weakness. Severe cases can cause respiratory depression requiring mechanical ventilation and quadriplegia [4]. The diagnosis is based on clinical presentation, cerebrospinal fluid (CSF) analysis, and electromyogram/nerve conduction studies (EMG/NCS). The CSF analysis will generally show normal pressure, few nucleated cells, and an elevated protein concentration [4]. The incidence of GBS is between 0.8 and 1.9 per 100,000 individuals annually, and it equally affects both genders [5]. The most common cause of GBS is infection (i.e., cytomegalovirus, Epstein-Barr virus, Campylobacter jejuni) [4]. Cases of GBS have also been associated with vaccination and systemic diseases such as Hodgkin’s lymphoma, sarcoidosis, and systemic lupus erythematosus [4]. The treatment of GBS is largely supportive; however, intravenous immunoglobulin (IVIG) and plasma exchange have been shown to shorten recovery time and oral steroids alone have not shown to be effective in GBS [6,7]. Overall mortality from GBS is between 3% and 7%; patients with multiple comorbidities, older age, and those requiring mechanical ventilation are at the highest risk [8]. The majority of deaths occur in more than 30 days from initial onset of symptoms [8]. Common causes of death include respiratory insufficiency, pulmonary infection, autonomic dysfunction, and cardiac arrest [8].

GBS has been linked to malignancy but is very rare and the mortality of GBS in patients with cancer is higher than the mortality of GBS in the general population [9]. Studies have shown the incidence of GBS is higher in patients with gastric adenocarcinoma, hepatocellular carcinoma, myelodysplastic syndrome, rectal carcinoma, angioimmunoblastic T-cell lymphoma, squamous cell carcinoma of the septum, breast cancer, colon cancer, and Hodgkin’s lymphoma [9,10]. In cases of Hodgkin’s lymphoma, IVIG as monotherapy did not prove as effective as in other situations and treatment of the underlying malignancy was essential [10]. In other cases, the administration of IVIG led to full recovery of GBS symptoms in patients with cancer [11,12].

## Case presentation

**Figure 1:**
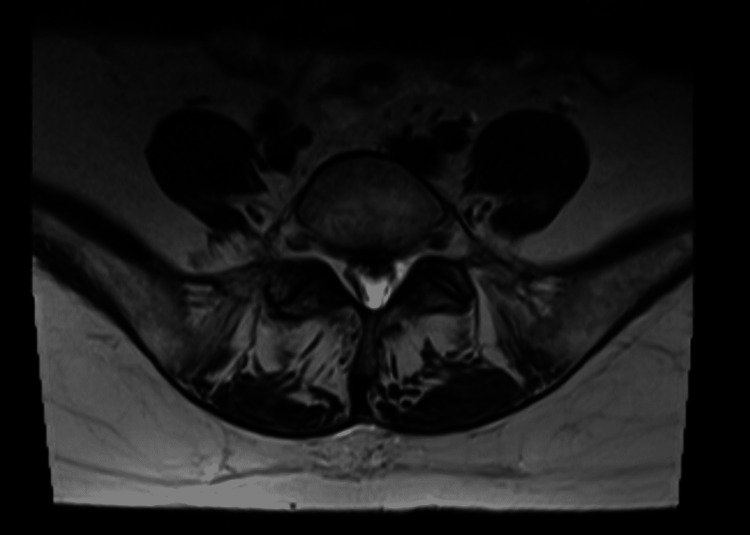
MRI of cervical spine showing chronic severe spinal canal stenosis at levels C4-C5 and C5-C6 secondary to degenerative change with spinal cord atrophy.

A 64-year-old Asian woman with moderately differentiated node-positive resected thoracic squamous cell carcinoma of the upper esophagus receiving 5-fluorouracil and cisplatin chemotherapy concurrently with radiation presented for two weeks of progressive lower extremity weakness and numbness. She had a past medical history including hypertension treated with amlodipine, but no risk factors for esophageal cancer such as tobacco use or excessive alcohol intake. She experienced acute worsening of symptoms 72 hours after cisplatin infusion, requiring walker four days after infusion, and wheelchair five days after infusion. She underwent an MRI (1.5 Tesla) with and without contrast of the cervical, thoracic, and lumbar spines that revealed chronic severe spinal canal stenosis at level C4-C5 and C5-C6 secondary to degenerative change with spinal cord atrophy, diffusely heterogeneous bone marrow signal without definitive focal bony mass, and no definite leptomeningeal disease (Figure 1).

EMG/NCS revealed length-dependent polyneuropathy with both axonal and demyelinating components. She underwent a lumbar puncture, which revealed findings consistent with albuminocytologic dissociation and increased CSF IgG. The patient's chemotherapy was held because of concern for chemotherapy toxicity, and she underwent two days of IVIG 2 g/kg infusions. She gradually demonstrated improvement in her weakness and paresthesias after treatment and was scheduled to receive an additional dose of IVIG approximately one month after her initial dose. She was discharged to an acute rehabilitation facility for further physical and occupational therapy.

## Discussion

ESCC has not been reported to cause peripheral or paraneoplastic neuropathy. Associated neurological paraneoplastic syndromes that have been reported include encephalitis, opsoclonus myoclonus syndrome, and necrotizing myelopathy [13-15]. There is a reported case of sensory motor polyneuropathy; however, this was reported in small cell carcinoma of the esophagus [16]. 

ESCC has been associated with neuropathy but in the context of dose-dependent platinum compounds, such as cisplatin and oxaliplatin [17]. Neuropathy induced by cisplatin is often pure sensory in a stocking-and-glove distribution and includes numbness, tingling, paresthesia, and neuropathic pain in both upper and lower extremities [17]. Motor and autonomic symptoms are rare with cisplatin. There have been no reported improvements in cisplatin-induced neuropathy with IVIG administration.

To our knowledge, this is the first case that reports a patient with ESCC developing GBS. A large case-control study in Denmark found the most common malignancies associated with GBS were lymphoma, lung, and prostate cancers [18]. Other malignancies that have been associated are nasal SCC, gastric adenocarcinoma, hepatocellular carcinoma, rectal carcinoma, and myelodysplastic syndrome [9]. One study with a total of 435 cases of GBS found only 13 of those patients also had a malignancy [19]. One case report of a patient who received treatment with oxaliplatin-based chemotherapy for the treatment of metastatic colon cancer developed GBS [11]. This patient responded to five days of IVIG and was able to make a full neurologic recovery [11]. Additional studies have supported the use of IVIG when treating GBS in patients who received chemotherapy for underlying malignancy [10,12]. Immune checkpoint inhibitors such as the CTLA-4 inhibitor ipilimumab, and the PD-1 inhibitor, nivolumab, have also been associated with cases of GBS [20]. These cases responded to IVIG and steroids as a treatment [20]. Overall, the incidence of GBS in cancer patients is rare.

## Conclusions

This case represents the first documented report of an association between ESCC and GBS. GBS is extremely rare in the general population and is much rarer in patients with cancer. In cases of GBS associated with most malignancies, patients respond to IVIG and have rapid improvement in their overall symptoms. Recognizing this phenomenon and treatment strategy is important to note to potentially help other patients in the future.
